# Supplementary figures and images for: Injection of prototypic celiac anti-transglutaminase 2 antibodies in mice does not cause enteropathy
Source: PLoS One. 2022 Apr 6;17(4):e0266543. doi: 10.1371/journal.pone.0266543 (PMC8985999; doi:10.1371/journal.pone.0266543)

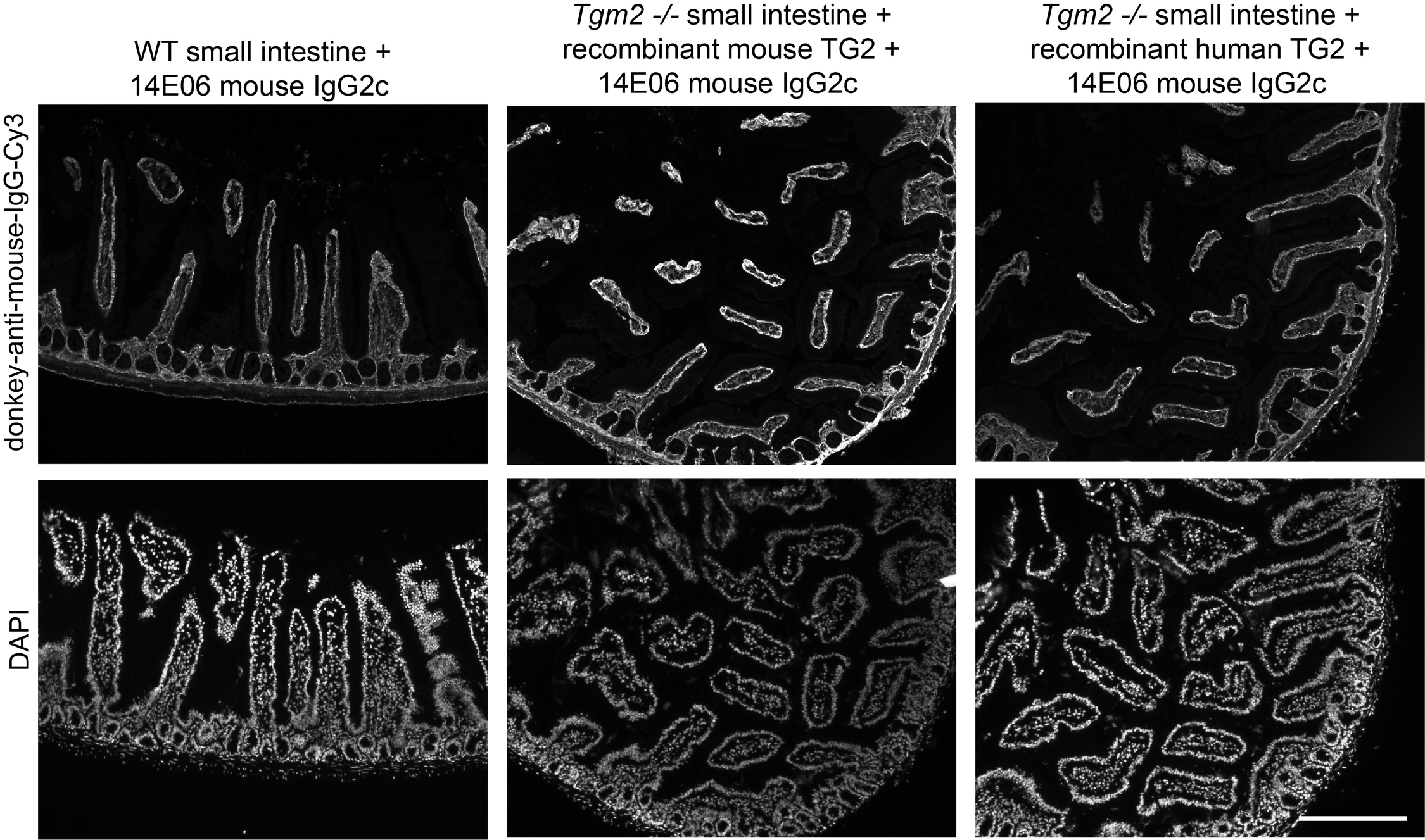

Supplement: S1 Fig — Hybridoma-derived 14E06 (mouse IgG2c) binds to endogenous TG2 in the ECM of mouse small intestine (left panel). Mouse 14E06 (IgG2c) also binds to recombinant human or mouse TG2 immobilized in the ECM of Tgm2-/- mouse small intestine (middle and right panel). Nuclei were counterstained with DAPI. Scale bar represents 100μm. (TIF) [file pone.0266543.s001.tif]

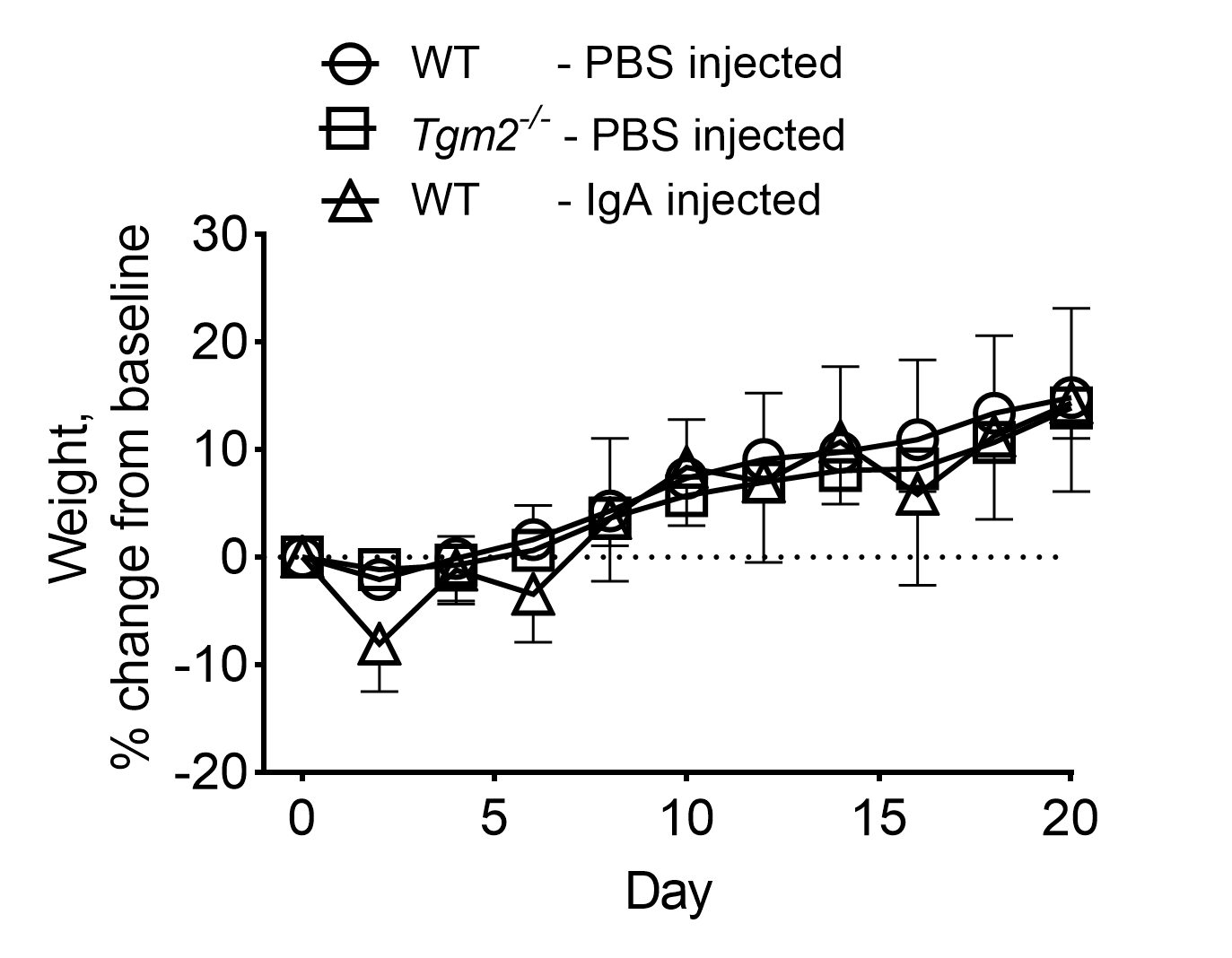

Supplement: S2 Fig — The graph reports weight as % change from baseline for the different groups as indicated. Dots and bars represent mean +/- SD. Data represent all mice of each group from the two independent experiments. (TIF) [file pone.0266543.s002.tif]
